# Novel exploration of Raman microscopy and non-linear optical imaging in adenomyosis

**DOI:** 10.3389/fmed.2022.969724

**Published:** 2022-10-20

**Authors:** Zhuowei Shen, Yingying He, Zhuoyi Shen, Xuefei Wang, Yang Wang, Zhengyu Hua, Nan Jiang, Zejiang Song, Rui Li, Zhen Xiao

**Affiliations:** ^1^Department of Obstetrics and Gynecology, First Affiliated Hospital of Dalian Medical University, Dalian, China; ^2^Department of Pathology, Dalian Medical Center for Women and Children, Dalian, China; ^3^Department of Information Science and Technology, Wenhua University, Wuhan, China; ^4^Department of Physics, Dalian University of Technology, Dalian, China; ^5^Department of Pathology, First Affiliated Hospital of Dalian Medical University, Dalian, China

**Keywords:** adenomyosis of uterus, disease diagnosis, Raman spectra, CARS, SHG

## Abstract

**Background:**

Adenomyosis is a common gynecological disease in women. A relevant literature search found that approximately 82% of patients with adenomyosis chose to undergo hysterectomy. However, women of childbearing age are more likely to undergo surgery to preserve the uterus. Because it is difficult to determine the extent of adenomyosis, it is almost impossible to resect adenomyotic tissue and retain the uterus at the same time.

**Materials and methods:**

Following ethics approval and patient consent, tissue samples were resected and prepared to create frozen slices for analysis. One slice was subjected to H&E staining while the remaining slices were photographed with Coherent Anti-Stokes Raman Scattering (CARS), Second-Harmonic Generation (SHG) microscopy, and Raman spectroscopy. Comparative observations and analyses at the same positions were carried out to explore the diagnostic ability of CARS, SHG, and Raman spectroscopy for adenomyosis.

**Results:**

In adenomyotic tissue, we found two characteristic peaks at 1,155 and 1,519 cm^–1^ in the Raman spectrum, which were significantly different from normal tissue. The substances shown in the CARS spectrum were represented by peaks of 1,519 cm^–1^. SHG microscopy showed a distribution of collagen at the focus of the adenomyosis.

**Conclusion:**

This study represents a novel analysis of Raman microscopy, CARS, and SHG in the analysis of adenomyotic lesions. We found the diffraction spectrum useful in determining the focal boundary and the diagnosis of adenomyosis in the tested samples.

## Introduction

Adenomyosis refers to the invasion of endometrial glands and stroma into the myometrium and the maintenance of functional changes such as periodic hyperplasia, exfoliation, and bleeding. The cause of the disease is unknown (1). It can lead to symptoms such as increased menstruation, prolonged menstruation, and progressive aggravated dysmenorrhea (2). A prior study by Di Donato and co-authors found that 21.8% of patients with endometriosis have adenomyosis. Patients with concurrent adenomyosis have been found to be older, and have a greater pain intensity and depth of infiltration of endometriosis (3). Furthermore, a diagnosis of adenomyosis in these patients has been shown to negatively impact postoperative pain following surgical treatment (4). Adenomyosis can be divided into two types: focal and diffuse. The uterus is uniformly enlarged in diffuse adenomyosis. Focal lesions, known as adenomyosis, grow locally and have no obvious boundary with surrounding tissue, which makes them difficult to resect during surgery. A population sample paper found that about 82% of patients with adenomyosis choose to undergo hysterectomy (5). However, total hysterectomy is obviously not feasible for women with reproductive needs. Uterine-sparing surgery is an alternative surgical treatment, but it is difficult to determine the scope and focus of adenomyosis as it is often mixed with the surrounding normal myometrium; it is therefore almost impossible to completely remove adenomyotic tissue while preserving the uterus (6). At present, the main methods of diagnosing adenomyosis are clinical symptoms and ultrasonography, and the gold standard of diagnosis is postoperative pathology, that is, H&E staining. However, H&E staining takes time and requires a pathologist. Identifying the lesion boundary to aid successful removal therefore represents a significant challenge for surgeons during uterine-sparing surgery (7).

Currently, utilizing engineering technology alongside medicine has proven popular. Among them, the application of optical microscopy in medicine is emerging. Coherent Anti-Stokes Raman scattering (CARS) is a third-order non-linear optical process based on the coherent excitation of molecular vibrations (8), which can obtain the molecular composition and distribution information of the sample to be tested according to the vibrational characteristics of the material molecules. Second-harmonic generation (SHG) microscopy has emerged as a powerful modality for imaging fibrillar collagen in a diverse range of tissues because it is highly sensitive to the collagen fibril/fiber structure (9).

Optical microscopy has been successfully applied to gastric cancer (8), colorectal cancer (9), human meningioma (10, 11), liver cancer (12), lung cancer (13), and other diseases. There have been a large number of studies on cervical cancer (14), ovarian cancer (15), endometrial carcinoma (16), and reproduction (17) in obstetrics and gynecology. Compared with the time-consuming traditional H&E staining method, the biggest advantage of optical microscopy lies in its convenience and efficiency. Notably, the prior application of Raman Microscopy in determining the lesion range in a cohort undergoing surgical treatment for brain cancer provided possible parallels to the identification and resection of tissue boundaries in adenomyosis patients (18). Our hypothesis therefore became: can the focus and scope of adenomyosis be determined through optical microscopy to meet the needs of women of childbearing age and to perform adenomyosis surgery with uterine preservation?

This study describes the first use of a Raman microscope, CARS, and SHG to study adenomyotic lesions.

## Materials and methods

### Sample preparation

This is a prospective study from February 2021 to March 2022. We randomly selected 10 patients (five normal and five with adenomyosis) who underwent surgery in the First Affiliated Hospital of Dalian Medical University. The adenomyosis patients were determined by preoperative ultrasound examination. After cutting off the uterus during the operation, we retain several pieces of tissue (in case of adenomyosis, parts of the adenomyosis lesion and the rest of normal muscle tissue will be retained) and place them in liquid nitrogen tanks for cold storage, in order to preserve the cell activity for the convenience of subsequent experiments. Patients with adenomyosis provided both adenomyotic and normal tissue samples while non-adenomyosis patients provided normal tissue samples. Finally, a total of 20 adenomyotic tissue samples and 20 normal samples (including five adenomyosis patients’ normal samples) were included in this study. Adenomyosis samples were from patients who underwent total hysterectomy due to adenomyosis, and normal *in vitro* samples of the control group were from normal uterine muscle tissues of patients who underwent total hysterectomy owing to non-malignant diseases (to prevent tumor tissues from affecting the results), such as hysteromyoma and uterine prolapse. All patients signed the informed consent form under the informed consent of the research process after surgery, allowing us to conduct experiments on their *in vitro* tissues. This experiment was certified by the ethics Association of the First Affiliated Hospital of Dalian Medical University (IRB number: PJ-KS-KY-2022-257).

After sample preparation, we took 40 fresh tissues (20 normal tissues and 20 adenomyosis tissues) about 1 × 1 cm in size from 10 uteruses (five normal tissues and five adenomyosis tissues), and made continuous frozen sections for each tissue. From each tissue, three 10 μm sections (slices) were cut. The three slices of the same tissue were numbered 1, 2, and 3. All “Slices 1” underwent H&E staining, and Slices 2 and 3 were directly observed under a non-linear optics microscope without any staining treatment.

### H&E

Two experienced pathologists observed all H&E-stained slices to provide a diagnosis of either adenomyosis or normal tissue; their corresponding Slices 2 and 3 were imaged by CARS and Raman microscope, respectively.

### Raman spectra

**FIGURE 1:**
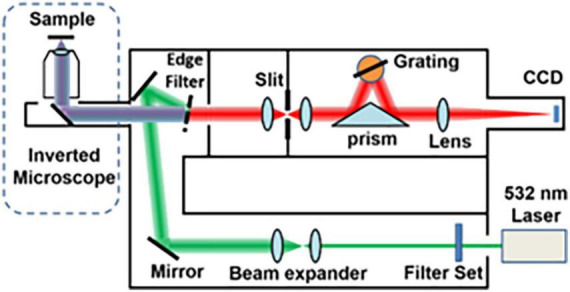
Layout of the optical system of the Raman micro-spectrometer.

Raman spectra were obtained using a commercial Raman micro-spectrometer (Renishaw, InVia system) at 532 nm excitation wave number, which was focused onto the muscles using a 50× (NA = 0.75) objective for an integration time of 10 s. Cosmic ray was removed after acquiring each spectrum using the Renishaw WiRE 4.4 software. The experimental setup and its schematic illustration are shown in Figure 1.

**FIGURE 2:**
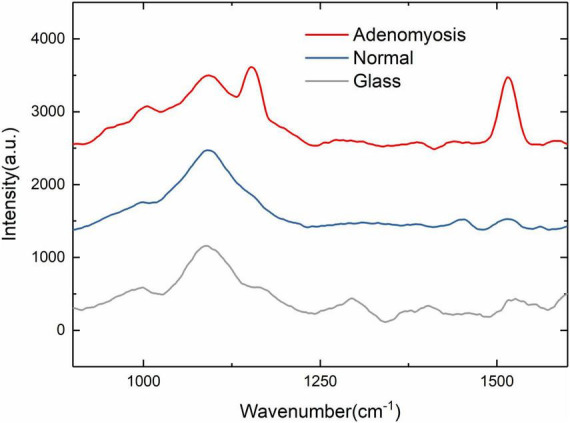
900–1,600 cm^– 1^ Raman spectra of normal, adenomyosis, and glass (normal, adenomyosis, and glass from top to bottom).

Because the Raman microscope displays spectrum images of substances in a limited range, different substances display different Raman signals, so the carrier glass carrying tissue slices will inevitably display their own Raman signals. At this time, the glass is measured separately to display the Raman signal of the glass itself as a reference (red in Figure 2), so that the peak value of the glass and the characteristic peak value of adenomyosis can be distinguished.

We first identified the characteristic wave number range in the range of 500–3,000 cm^–1^. As shown in Figure 2, the characteristic wave number is about 1,200 and 1,500 cm^–1^, so we set the wave number range at 900–1,600 cm^–1^ to facilitate the experiment.

### Anti-stokes Raman scattering and second-harmonic generation

**FIGURE 3:**
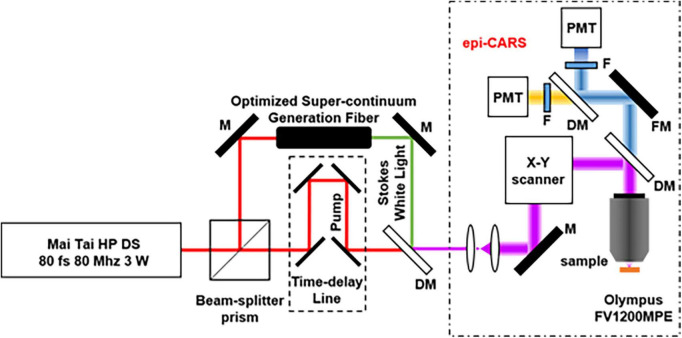
Optical path of the CARS system (M, mirror; DM, Dichroic Mirrors; F, Filter; FM, Flip Mirror; PMT, photomultiplier tube).

Figure 3 shows a schematic of the CARS system for non-linear optical imaging. Briefly, a mode-locked 80 fs Ti:sapphire laser (MaiTai, Spectra Physics, Santa Clara, USA) is tuned to 800 nm with pulse width at an 80 MHz repetition rate and divided into two parts by a polarization beam splitter. One beam works as the pump beam; the other beam is used to pump a photonic crystal fiber to produce the Stokes beam for CARS imaging. Two beams are combined at the dichroic mirror. The combined beams are sent into a multiphoton scanning microscope (Olympus, FV1200) and focused on the sample by an objective (10×, NA 0.4; UplanApo, Olympus, Tokyo, Japan). The average power of 75 mW is used for the pump and the probe beam. The CARS and SHG signals pass through a bandpass filter, respectively, before being detected by the PMT.

## Results

**FIGURE 4:**
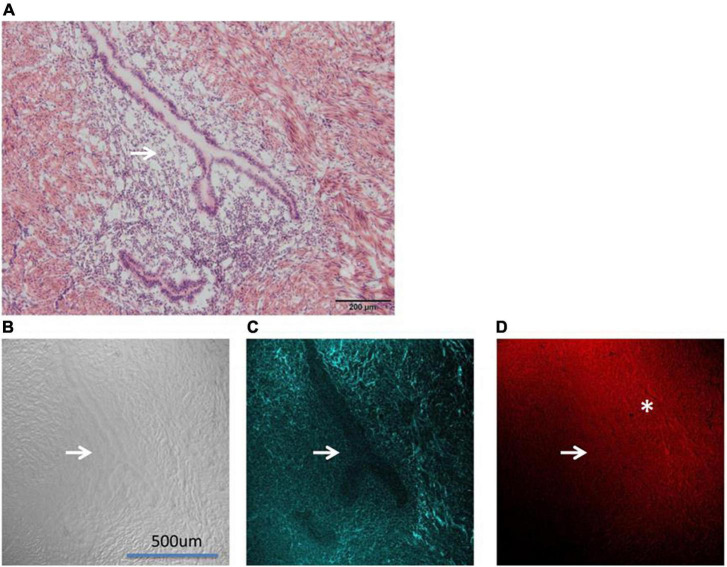
Shows the images of adenomyosis lesions under different microscopes (→: Ectopic uterine gland). **(A)** An H&E staining section. **(B)** DIC (differential interference contrast microscope) imaging. **(C)** SHG imaging. **(D)** CARS imaging (*: fibro-collagen proliferation).

All Slice 1 samples were viewed under the microscope before imaging for records (Figure 4A). Since Slices Nos. 1, 2, and 3 were cut continuously by a slicer, any differences can be ignored. We observed images with CARS and SHG at the same position of Slices 2. Examples of images are adenomyosis lesions imaged by CARS and SHG (Figure 4).

Figure 4A shows the contrast diagram of H&E staining. The lesions shown in the figure are the subject of this study. The “Y” structure pointed by the white arrow in the figure is the ectopic endometrial structure in the myometrium, namely, the uterine gland. As shown in Figure 4A, the uterine gland is a single tube gland with branches at its end, mainly composed of secretory cells.

Figure 4B shows the differential interference contrast microscope (DIC imaging). DIC imaging is an image directly observed by the naked eye without staining. From the DIC imaging, we can see the sense of uneven layers in the image, vaguely seeing the ectopic uterine gland (the position indicated by the white arrow), but the peripheral structure is not clear.

In the Figure 4C CARS microscope presents the outline of the ectopic uterine gland perfectly. It can be seen that under the CARS microscope, the glandular part of the uterine gland is not imaged (the position indicated by the white arrow), and the image intensity of the surrounding interstitial part is relatively light, while the intensity of the surrounding muscle layer is relatively high. We used a 720 nm filter in CARS. The Raman wave number range corresponding to the 720 nm filter just includes our second characteristic peak of 1,519 cm^–1^. Therefore, we determined that the imaging substance of CARS was consistent with the representative substance of the second characteristic peak in Raman imaging.

Figure 4D shows the SHG image. Interestingly, SHG, unlike CARS imaging, showed stronger intensity in the uterine gland and its surrounding fibro-collagen proliferation section. The SHG microscope is widely used to image various fibrous collagens. 3D also clearly shows the distribution of collagen around the lesion. It can be seen that fibro-collagen proliferation exists around the uterine gland (*Marking section).

In the range of 900–1,600 cm^–1^, the characteristic wave number of adenomyosis is more obvious and allows a clear distinction between the Raman spectrum curve of normal tissue and adenomyosis (Figure 2). In the following experiment, we used adenomyosis tissues from different patients, and the characteristic peaks appeared at 1,155 and 1,519 cm^–1^.

## Discussion

In this report, we used Raman, DIC, CARS, and SHG microscopes to directly image tissue sections without staining, and took HE staining images at the same location for comparison.

CARS microscopy, probing vibrations of molecular bonds for image contrast, and the high vibrational Raman cross sections of many hydrogen carbon bonds make the technique suitable for imaging polymers (19). CARS microscopy derives its contrast from intrinsic molecular vibrations in a sample; the CH group of membrane and cortical cytoskeleton proteins are the basis of CARS imaging (20). In the absence of staining (Figure 4C), CARS can clearly show the outline of the ectopic uterine gland and its boundary with surrounding tissue structure compare with Figure 4A, which has been stained with H&E. SHG visualizes highly ordered tissue structures, which are non-centrosymmetric like type I collagen fibers (21). As shown in Figure 4D, there is obvious fibro-collagen proliferation around the uterine gland, which is caused by bleeding of adenomyosis (22).

According to previous experiments, the characteristic curve of cervical cancer is concentrated at 720, 785, 1,095, 1,258, and 1,579 cm^–1^ (23–25). This is different from the representative peaks of adenomyosis (1,155 and 1,519 cm^–1^) found in our study. Most studies regarding Raman microscopy in adenomyosis focus on the serological aspects of patients (26). During our literature search, only one such direct histological study was found. In this article (27), Wang et al. identified a peak different from that of normal tissue at 1,173 cm^–1^ in adenomyosis, believing the peak is induced by delta (C—O) shifts. Our initial peak was found to be 1,155 cm^–1^. Considering Wang and co-authors used a light source of 785 nm compared to our own source at 532 nm, we consider that this finding is broadly consistent. However, our finding of the additional peak at 1,519 cm^–1^ in adenomyosis samples represents a novel finding. To understand our novel finding, we reviewed the existing literature to identify biological macromolecules and concurrent Raman wave numbers (Table 1). First, after an extensive literature search and integration, we created a corresponding table between Raman wave numbers and biological macromolecules (14, 16, 28–30). From Table 1, we can see that most representative substances with similar wave numbers are the same (however, there may be errors caused by different measurements). The corresponding substance of 1,516 cm^–1^ is amide II, considering some errors caused by different experimental conditions (temperature, tissue freshness, etc.) and instrument measurements, so our first hypothesis about the characteristic peak at 1,519 cm^–1^ was amide II. Of particular interest to our findings, two prior lung cancer studies using Raman microscopy found characteristic carotenoid Raman peaks at 1,152 and 1,518 cm^–1^ with the Raman peaks in lung cancer patients lower than those in normal subjects. The authors suggested these findings reflected C–C and conjugated C=C bond stretch (24, 31). In our study, characteristic peaks were found at 1,155 and 1,519 cm^–1^ in adenomyosis tissue, which is very similar to the characteristic peaks of carotenoids at 1,152 and 1,518 cm^–1^ in the previous two studies. Carotenoids represent the main source of Vitamin A in the body and provide anti-oxidation, immune regulation, anti-cancer, and anti-aging effects. Our findings representing similar peaks may support a possible relationship between carotenoids and adenomyosis; however, this remains speculative and requires additional investigation.

**TABLE 1:** Wave number of biomacromolecules.

Assignment	Raman shift (cm^–1^)
DNA	481, 784, 788, 826
DNA/RNA	1,231, 1,320
Saccharides	1,370
Monosaccharide	898
Disaccharide	898
Polysaccharide	477
Glycogen	933, 1,003, 1,025, 1,150
Amylaceum	540
Collagens	859, 1,032, 1,303, 1,309, 1,325, 1,332, 1,339, 1,445
Phosphatidylinositol	415, 519, 576
Phospholipid	1,085, 1,032, 1,078, 1,445, 1,745
Cholesterol	548
Cholesteryl ester	538, 614
Lipid	877, 968, 1,125, 1,057, 1,060, 1,095, 1,124, 1,275, 1,309, 1,369, 1,437, 1,447, 1,450, 1,452
Glycerol	630
Nuclein	1,299, 1,340, 1,578
Tyrosine	640, 642, 643, 821, 823, 830, 835, 849, 853, 855, 859, 1,170, 1,616
Methionine	695
Aspartate	1,700
Glutamate	1,700
Tryptophan	745, 752, 758, 880, 1,208, 1,365, 1,374, 1,376, 1,552, 1,560, 1,561, 1,616, 1,618, 1,618
Proline	814, 821, 853, 855, 880, 918, 928, 933, 935, 936, 1,043, 1,066, 1,447
Hydroxyproline	821, 853, 876, 1,588
Valine	928, 933, 935, 936, 1,066
Phenylalanine	1,000, 1,002, 1,003, 1,004, 1,030, 1,104, 1,582, 1,583, 1,588, 1,602
Cysteine	495–516
Protein	933, 951, 1,158, 1,369
Phosphorylated protein	968, 970
Pyrimidine ring	766
Uracil	780, 784
Cytosine	784, 1,175, 1,290, 1,506
Thymine	784
Guanine	1,175, 1,369
Adenine	721, 1,335
Porphyrin	1,369
C-C skeleton	928, 938, 1,130, 1,561
C-C stretching (collagen)	817
C-C stretching (phenylalanine)	1,339
C-H stretching (protein)	1,295
C-N stretching (protein)	1,053, 1,128
C-O stretching (protein)	1,053
C-O stretching (lipid)	1,723, 1,738, 1,792
Ribose vibration	867, 915
Antisymmetric vibration of phosphoric acid	1,185–300
Antisymmetric phosphate stretching vibration	1,230
Amide I	1,600, 1,601, 1,624, 1,637, 1,640, 1,645, 1,654, 1,655, 1,658, 1,660, 1,664, 1,670, 1,685, 1,697
Amide II	1,516, 1,570
Amide III	1,234, 1,236, 1,243, 1,246, 1,255, 1,275, 1,285, 1,302
β-Carotenoids	1,152, 1,518, 1,520

The outstanding advantages of Raman spectroscopy lie in its label-free nature and timeliness, which reduce the waiting time of intraoperative pathology and the burden upon pathologists at the surgery. Currently, Hand-Held Raman technology has been successfully applied to detect air components and diagnose plant diseases (32–34). There are also a large number of intraoperative boundary studies of brain tumors in medicine (18). Currently, there is no research regarding Hand-Held Raman technology on disease or surgery in obstetrics and gynecology. Our results suggest a possible further role for Hand-Held Raman microscopy in assisting the intraoperative diagnosis of adenomyosis and the localization of lesion boundaries to improve potential surgical outcomes in patients. Similarly, handheld SHG technology are also areas that have not been studied and discussed. The results of this study also found the potential utility in determining the location of adenomyosis lesions. SHG also confirmed the proliferation of fibro-collagen caused by bleeding around adenomyosis lesions. The application of these two microscopes in surgery will further help to determine and diagnose the location of adenomyosis lesions.

## Conclusion

In this experiment, a Raman microscope, CARS, and SHG were used to study adenomyosis, which demonstrated the role of non-linear optics in diagnosing adenomyosis and distinguishing lesion boundaries. Moreover, the combination of CARS and SHG microscopes produces more extensive and complementary information.

## Data availability statement

The original contributions presented in this study are included in the article/supplementary material, further inquiries can be directed to the corresponding authors.

## Ethics statement

The studies involving human participants were reviewed and approved by the Ethics Department of the First Affiliated Hospital of Dalian Medical University. The patients/participants provided their written informed consent to participate in this study.

## Author contributions

ZWS wrote the article, designed the experiment, and analyzed the results. YH, XW, ZH, and NJ were four experienced pathology teachers who contributed to our H&E staining and sectioning. ZYS, YW, and ZJS contributed to the revision of charts, literature retrieval, and experiments. This article had taken place under the guidance of two experienced tutors who were corresponding authors of this article, ZX and RL. All authors agree to be responsible for the content of this article and the submitted version.

## References

[1] Camboni A Marbaix E. Ectopic endometrium: the pathologist’s perspective. *Int J Mol Sci.* (2021) 22:10974. 10.3390/ijms222010974 PMC8540175 34681634

[2] Szubert M Kozirog E Wilczynski J. Adenomyosis as a risk factor for myometrial or endometrial neoplasms-review. *Int J Environ Res Public Health.* (2022) 19:2294. 10.3390/ijerph19042294 PMC8872164 35206475

[3] Di Donato N Montanari G Benfenati A Leonardi D Bertoldo V Monti G Prevalence of adenomyosis in women undergoing surgery for endometriosis. *Eur J Obstet Gynecol Reprod Biol.* (2014) 181:289–93. 25201608 10.1016/j.ejogrb.2014.08.016

[4] Brosens I Benagiano G. Poor results after surgery for rectovaginal endometriosis can be related to uterine adenomyosis. *Hum Reprod.* (2012) 27: 3360–1. 22814482 10.1093/humrep/des276

[5] Yu O Schulze-Rath R Grafton J Hansen K Scholes D Reed SD. Adenomyosis incidence, prevalence and treatment: United States population-based study 2006-2015. *Am J Obstet Gynecol.* (2020) 223:94 e1–e10. 10.1016/j.ajog.2020.01.016 31954156

[6] Stratopoulou CA Donnez J Dolmans MM. Conservative management of uterine adenomyosis: medical vs. surgical approach. *J Clin Med.* (2021) 10:4878. 10.3390/jcm10214878 PMC8584979 34768397

[7] Horng HC Chen CH Chen CY Tsui KH Liu WM Wang PH. Uterine-sparing surgery for adenomyosis and/or adenomyoma. *Taiwan J Obstet Gynecol.* (2014) 53:3–7. 24767637 10.1016/j.tjog.2014.01.001

[8] Allen CH Hansson B Raiche-Tanner O Murugkar S. Coherent anti-Stokes Raman scattering imaging using silicon photomultipliers. *Opt Lett.* (2020) 45:2299–302. 10.1364/OL.390050 32287218

[9] Chen X Nadiarynkh O Plotnikov S Campagnola PJ. Second harmonic generation microscopy for quantitative analysis of collagen fibrillar structure. *Nat Protoc.* (2012) 7:654–69. 22402635 10.1038/nprot.2012.009 PMC4337962

[10] Zhang L Zhou Y Wu B Zhang S Zhu K Liu CH Intraoperative detection of human meningioma using a handheld visible resonance Raman analyzer. *Lasers Med Sci.* (2022) 37:1311–9. 10.1007/s10103-021-03390-2 34365551

[11] Hollon T Orringer DA. Label-free brain tumor imaging using Raman-Based methods. *J Neurooncol.* (2021) 151:393–402. 10.1007/s11060-019-03380-z 33611706 PMC9333091

[12] Yan S Cui S Ke K Zhao B Liu X Yue S Hyperspectral stimulated Raman scattering microscopy unravels aberrant accumulation of saturated fat in human liver cancer. *Anal Chem.* (2018) 90:6362–6. 10.1021/acs.analchem.8b01312 29757615

[13] Zhang Z Yu W Wang J Luo D Qiao X Qin X Ultrasensitive Surface-Enhanced Raman scattering sensor of gaseous aldehydes as biomarkers of lung cancer on dendritic Ag nanocrystals. *Anal Chem.* (2017) 89:1416–20. 10.1021/acs.analchem.6b05117 28208308

[14] Karunakaran K Saritha VN Joseph MM Nair JB Saranya G Raghu KG Diagnostic spectro-cytology revealing differential recognition of cervical cancer lesions by label-free surface enhanced Raman fingerprints and chemometrics. *Nanomedicine.* (2020) 29:102276. 10.1016/j.nano.2020.102276 32736038

[15] Honda K Hishiki T Yamamoto S Yamamoto T Miura N Kubo A On-tissue polysulfide visualization by surface-enhanced Raman spectroscopy benefits patients with ovarian cancer to predict Post-Operative chemosensitivity. *Redox Biol.* (2021) 41:101926. 10.1016/j.redox.2021.101926 PMC8010883 33752108

[16] Schiemer R Furniss D Phang S Seddon AB Atiomo W Gajjar KB. Vibrational biospectroscopy: an alternative approach to endometrial cancer diagnosis and screening. *Int J Mol Sci.* (2022) 23:4859. 10.3390/ijms23094859 35563249 PMC9102412

[17] O’Brien CM Vargis E Rudin A Slaughter JC Thomas G Newton JM In vivo Raman spectroscopy for biochemical monitoring of the human cervix throughout pregnancy. *Am J Obstet Gynecol.* (2018) 218:528 e1–e18. 29410109 10.1016/j.ajog.2018.01.030 PMC5916496

[18] Karabeber H Huang R Iacono P Samii JM Pitter K Holland EC Guiding brain tumor resection using surface-enhanced Raman scattering nanoparticles and a Hand-Held Raman scanner. *ACS Nano.* (2014) 8:9755–66. 10.1021/nn503948b 25093240 PMC4212801

[19] Kee TW Cicerone MT. Simple approach to one-laser, broadband coherent Anti-Stokes Raman scattering microscopy. *Opt Lett.* (2004) 29:2701–3. 10.1364/ol.29.002701 15605477 PMC4045474

[20] Arkill KP Moger J Winlove CP. The structure and mechanical properties of collecting lymphatic vessels: an investigation using multimodal nonlinear microscopy. *J Anat.* (2010) 216:547–55. 10.1111/j.1469-7580.2010.01215.x 20345855 PMC2871990

[21] Sehm T Uckermann O Galli R Meinhardt M Rickelt E Krex D Label-free multiphoton microscopy as a tool to investigate alterations of cerebral aneurysms. *Sci Rep.* (2020) 10:12359. 10.1038/s41598-020-69222-5 32704100 PMC7378195

[22] Wang S Li B Duan H Wang Y Shen X Dong Q Abnormal expression of connective tissue growth factor and its correlation with fibrogenesis in adenomyosis. *Reprod Biomed Online.* (2021) 42:651–60. 10.1016/j.rbmo.2020.11.002 33431336

[23] Daniel A Prakasarao A Ganesan S. Near-infrared Raman spectroscopy for estimating biochemical changes associated with different pathological conditions of cervix. *Spectrochim Acta A Mol Biomol Spectrosc.* (2018) 190:409–16. 10.1016/j.saa.2017.09.014 28954253

[24] Huang Z McWilliams A Lui H McLean DI Lam S Zeng H Near-infrared Raman spectroscopy for optical diagnosis of lung cancer. *Int J Cancer.* (2003) 107:1047–52. 14601068 10.1002/ijc.11500

[25] Lyng FM Faolain EO Conroy J Meade AD Knief P Duffy B Vibrational spectroscopy for cervical cancer pathology, from biochemical analysis to diagnostic tool. *Exp Mol Pathol.* (2007) 82:121–9. 10.1016/j.yexmp.2007.01.001 17320864

[26] Parlatan U Inanc MT Ozgor BY Oral E Bastu E Unlu MB Raman spectroscopy as a non-invasive diagnostic technique for endometriosis. *Sci Rep.* (2019) 9:19795. 10.1038/s41598-019-56308-y 31875014 PMC6930314

[27] Liu G Liu JH Zhang L Yu F Sun SZ. [Raman spectroscopic study of uterine pathological tissue]. *Guang Pu Xue Yu Guang Pu Fen Xi.* (2005) 5:723–5. 16128073

[28] Duraipandian S Mo J Zheng W Huang Z. Near-infrared Raman spectroscopy for assessing biochemical changes of cervical tissue associated with precarcinogenic transformation. *Analyst.* (2014) 139:5379–86. 10.1039/c4an00795f 25140756

[29] Sitarz K Czamara K Bialecka J Klimek M Zawilinska B Szostek S HPV infection significantly accelerates glycogen metabolism in cervical cells with large nuclei: Raman microscopic study with subcellular resolution. *Int J Mol Sci.* (2020) 21:2667. 10.3390/ijms21082667 32290479 PMC7215571

[30] Wang J Zheng CX Ma CL Zheng XX Lv XY Lv G-D Raman spectroscopic study of cervical precancerous lesions and cervical cancer. *Lasers Med Sci.* (2021) 36:1855–64. 33404885 10.1007/s10103-020-03218-5 PMC8594213

[31] Bakker Schut TC Puppels GJ Kraan YM Greve J van der Maas LL Figdor CG. Intracellular carotenoid levels measured by Raman micro spectroscopy: comparison of lymphocytes from lung cancer patients and healthy individuals. *Int J Cancer.* (1997) 74:20–5. 10.1002/(sici)1097-0215(19970220)74:1<20::aid-ijc4>3.0.co;2-2 9036864

[32] Egging V Nguyen J Kurouski D. Detection and identification of fungal infections in intact wheat and sorghum grain using a hand-held Raman spectrometer. *Anal Chem.* (2018) 90:8616–21. 10.1021/acs.analchem.8b01863 29898358

[33] Farber C Sanchez L Kurouski D. Confirmatory non-invasive and non-destructive identification of poison ivy using a hand-held Raman spectrometer. *RSC Adv.* (2020) 10:21530–4. 10.1039/d0ra03697h 35518747 PMC9054379

[34] Heleg-Shabtai V Zaltsman A Sharon M Sharabi H Nir I Marder D Explosive vapour/particles detection using SERS substrates and a hand-held Raman detector. *RSC Adv.* (2021) 11:26029–36. 10.1039/d1ra04637c 35479444 PMC9037225

